# Racial-ethnic disparities in concurrent rates of peripapillary & macular OCT parameters among a large glaucomatous clinical population

**DOI:** 10.1038/s41433-024-03103-3

**Published:** 2024-05-04

**Authors:** Gabriele Gallo Afflitto, Swarup S. Swaminathan

**Affiliations:** 1 Bascom Palmer Eye Institute, University of Miami Miller School of Medicine, Miami, FL USA; 2 Ophthalmology Unit, Department of Experimental Medicine, Università di Roma “Tor Vergata”, Rome, Italy

**Keywords:** Glaucoma, Diagnosis, Epidemiology

## Abstract

**Objectives:**

To compare rates of change in peripapillary retinal nerve fibre layer (pRNFL) and macular ganglion cell-inner plexiform layer (mGCIPL) parameters among different race-ethnicities from a large electronic health record database of subjects with or suspected of glaucoma.

**Methods:**

In this retrospective cohort study, rates of change were obtained using joint longitudinal linear mixed models for eyes with ≥3 visits and ≥1 year of follow-up, adjusting for age, sex, intraocular pressure, central corneal thickness, and baseline pRNFL and mGCIPL thickness. Best linear unbiased predictor estimates of various parameters were stratified by baseline glaucoma severity and analysed by racial-ethnic group.

**Results:**

A total of 21,472 spectral domain optical coherence tomography (OCT) pRNFL scans and 14,431 mGCIPL scans from 2002 eyes were evaluated. A total of 200 (15.6%) and 601 (46.8%) subjects identified as non-Hispanic Black (NHB) and Hispanic, respectively. NHB eyes exhibited faster rates of change in pRNFL among glaucoma suspect (global pRNFL −0.57 ± 0.55 µm/year vs. −0.37 ± 0.62 µm/year among Hispanics, *p* < 0.001), mild glaucoma (superior pRNFL quadrant −1.20 ± 1.06 µm/year vs. −0.75 ± 1.51 µm/year among non-Hispanic Whites (NHW), *p* = 0.043), and moderate glaucoma eyes (superior pRNFL quadrant −1.31 ± 1.49 µm/year vs. −0.52 ± 1.26 µm/year among Hispanics, *p* = 0.003). NHB eyes exhibited faster rates of mGCIPL loss corresponding to pRNFL rates. Global pRNFL and mGCIPL rates were strongly correlated (R^2^ = 0.70).

**Conclusions:**

Adjusted rates of pRNFL and mGCIPL loss significantly differed between racial-ethnic groups when stratified by glaucoma severity, with faster rates among NHB patients. These differences highlight key racial-ethnic disparities in adjusted rates of glaucoma OCT parameters.

## Introduction

Glaucoma is a progressive, multifactorial optic neuropathy accompanied by characteristic structural changes and functional visual field loss [1, 2]. It is estimated that nearly 70 million people have been diagnosed with the disease, a number which is expected to increase [3]. Diagnosis and management of glaucoma rely on the detection of disease progression, commonly assessed using standard automated perimetry (SAP) and optical coherence tomography (OCT) [4, 5]. SAP has been used as the main or secondary outcome measures of disease progression in major glaucoma treatment trials [6, 7]. However, structural changes typically precede SAP abnormalities [8]. OCT provides an objective analysis of structural damage to the peripapillary retinal nerve fibre layer (pRNFL) and inner retina in the macula to assess glaucomatous progression. The latter has been described by various parameters such as the ganglion cell layer, ganglion cell complex, and macular ganglion cell-inner plexiform layer (mGCIPL) [9, 10].

Several prospective and retrospective studies have described pRNFL and inner retinal changes in glaucoma patients [9–13]. However, most have focused on smaller populations, suggesting the value of analysing larger clinical databases of patients receiving routine care [9–14]. The lack of racial and ethnic diversity in prospective studies restricts their applicability to heterogenous clinical populations [15]. In addition, studies have reported that individuals of African descent have a higher risk of visual impairment from glaucoma compared to those of European descent [16]. However, potential differences in rates of change in OCT parameters between different racial-ethnic groups have not been investigated using a large population receiving routine care.

The purpose of this study was to characterise rates of change in pRNFL and mGCIPL OCT parameters from a large database of glaucoma patients derived from an electronic health record (EHR) system of a tertiary eye care institution that cares for a diverse racial-ethnic population. We aimed to evaluate how jointly modelled rates of pRNFL and mGCIPL parameters differed between racial-ethnic groups when stratified by baseline disease severity and adjusted for key ocular and demographic characteristics.

## Methods

The University of Miami institutional review board approved this study (protocol #20191100). However, a waiver for obtaining informed consent was granted due to the retrospective nature of this research. The study adhered to the Declaration of Helsinki and the Health Insurance Portability and Accountability Act.

### Inclusion & exclusion criteria

This analysis was a retrospective study of patients from the Bascom Palmer Glaucoma Repository (BPGR). This database contains demographic, exam, imaging, and procedural data of eyes with glaucoma or suspected of having glaucoma examined at the clinical sites of the Bascom Palmer Eye Institute (BPEI), identified using International Classification of Diseases (ICD) codes (Supplementary Table 1) from the electronic health record (EHR) system (Epic Systems, Verona, WI). Patients were all aged 18 years or older at the time of first visit. The BPGR contains data from over 70,000 patients. Tests from any eyes that were diagnosed with exclusionary diagnoses (Supplementary Table 2) were excluded if they followed the first date of any of these diagnoses. All pRNFL and mGCIPL data using the Zeiss Cirrus spectral-domain OCT system were extracted from Zeiss Forum (Carl Zeiss Meditech, Dublin, CA). OCT data were available beginning April 2008. All data available as of February 1, 2022 were collected. For this study, we extracted OCT, demographic, central corneal thickness (CCT), and intraocular pressure (IOP) data from the BPGR.

### OCT data preparation

OCT scans were required to have a signal strength of 7 of 10 or greater. Any pRNFL OCT scans with global pRNFL values less than 30 µm or greater than 130 µm were eliminated as similarly completed in prior studies [17]. Based on the expected range of global mGCIPL values [18, 19], mGCIPL OCT scans with global thickness values greater than 105 µm and less than 33 µm were eliminated. In addition, any mGCIPL scans with “xxx” assigned to any sector values were eliminated. Based on the pRNFL “floor” of the Cirrus OCT system [20], eyes that had a global pRNFL thickness <57 µm at baseline were eliminated. Any tests that followed glaucoma surgery (trabeculectomy, aqueous shunt insertion, cyclophotocoagulation – identified by Current Procedural Terminology (CPT) codes 66710, 66172, 0192 T, 66183, 66179, 66180, 66710, and 66711) were excluded.

Subjects were required to have ≥ 3 visits with high-quality pRNFL and mGCIPL OCT scans over a minimum of 12 months of follow-up, following the study design of a recent large population study [17]. Only those pRNFL OCT scans that were performed within the same period as the mGCIPL scans were included to evaluate concurrent rates. Only those eyes that had CCT measurements, IOP data, and a 24-2 visual field with false positives < 15% and fixation losses < 33% within 12 months of the first RNFL scan were included. This baseline visual field was used to stage the eye using Hodapp-Anderson-Parrish (HAP) criteria as suspect, mild, moderate, or severe as previously completed [17, 21].

### Statistical analysis

We modelled rates of change in pRNFL and mGCIPL parameters using joint longitudinal linear mixed models. Linear mixed modelling has been used extensively in glaucoma research by various groups [5, 14, 21–23]. In brief, these models provide improved estimates of rates of change compared to traditional ordinary least squares regression by borrowing strength from population data; eyes with fewer tests borrow strength from the population. These models estimate the average rate of change of a predictor variable as a function of time and includes subject and eye-specific random effects, which function as adjustments to provide unique estimates for each eye. Joint longitudinal modelling has been utilised previously in glaucoma research to model related outcome measures [24, 25]. This approach leads to a shared distribution of random effects for the two predictor variables, thereby reducing the degree of measurement error and thus allowing for an improved assessment of the true relationship between outcome variables [8].

In this study, we modelled anatomically related pRNFL and mGCIPL parameters together in separate joint longitudinal mixed models: the superior pRNFL quadrant was modelled with superior and superotemporal mGCIPL sectors, the inferior pRNFL quadrant was modelled with inferior and inferotemporal mGCIPL sectors, and the temporal pRNFL quadrant was modelled with superonasal and inferonasal mGCIPL sectors. In an alternate model, superonasal and inferonasal mGCIPL sectors were modelled with the superior and inferior pRNFL quadrants respectively, but results were noted to be similar to the original models. Given the lack of an anatomic relationship between macular OCT parameters and the nasal pRNFL quadrant, the latter was modelled alone in a traditional linear mixed model. With respect to global indices, we modelled global pRNFL with global and minimum mGCIPL values. Estimates of rates of change for each of these parameters for individual eyes were obtained from best linear unbiased prediction (BLUP) [7, 25]. Of note, we accounted for inter-eye correlations by clustering measurements by eye. More complex models including nesting within patient did not improve model performance as assessed by Bayesian information criterion and BLUP estimates, and thus the simpler models were utilised.

Extracted demographics included date of birth, sex, self-reported race, and self-reported ethnicity. Age was calculated at the date of the first visit. In accordance with the study design of a recent large population study, patients were grouped as Non-Hispanic White (NHW), Non-Hispanic Black (NHB), Hispanic, or Other based on self-report [26]. Patients that did not identify with the first three categories or declined to answer were classified as Other. Models adjusted for age, sex, CCT, mean IOP, and baseline pRNFL and mGCIPL thickness, which were centred on their respective population means. Only IOP data between the first and last OCT scans were used to calculate mean IOP. Statistical comparisons between rates of racial-ethnic groups were completed using the Kruskal-Wallis rank sum test. Pairwise comparisons were completed using Dunn test with Bonferroni correction for multiple comparisons. *P*-values presented have been adjusted for multiple comparisons. All analyses were completed in R version 4.2.2 (R Foundation for Statistical Computing, Vienna, Austria).

## Results

A total of 2002 eyes from 1284 subjects (mean age 65.9 ± 11.8 years) were included in the study. Most subjects were female (61.8%), and 423 (32.9%) subjects identified as NHW, 200 (15.6%) as NHB, 601 (46.8%) as Hispanic, and 60 (4.7%) as Other (Table 1). Overall, 21,472 OCT pRNFL and 14,431 OCT mGCIPL scans were analysed. Table 2 summarises eye-level characteristics of the cohort, classified by glaucoma severity and racial-ethnic group. Of the 2002 eyes, 1,118 (55.8%) eyes had a normal visual field (and thus were labelled as “glaucoma suspect” in this study), 440 (22.0%) eyes were classified as having mild disease, 237 eyes (11.8%) were classified as moderate, and 207 eyes (10.3%) were identified as having severe glaucoma at baseline. Statistically significant differences (*p* < 0.001) were observed among the groups for baseline SAP MD, pRNFL thickness, mGCIPL thickness, CCT, and mean IOP (Table 2). However, the effect sizes of these differences were relatively small except for CCT. Follow-up time (mean 3.2 ± 1.0 years) and number of visits per patient (mean 3.8 ± 0.9 visits) were similar between groups (*p* = 0.13 and *p* = 0.40 respectively).

**Table 1:** Patient demographics.

Characteristic	Overall, *N* = 1284^a^	Non-Hispanic White, *N* = 423^a^	Non-Hispanic Black, *N* = 200^a^	Hispanic, *N* = 601^a^	Other, *N* = 60^a^	*p*-value^b^
Age	65.9 ± 11.8 66.6 (59.5, 74.1)	68.9 ± 11.4 70.8 (63.9, 76.4)	63.0 ± 10.3 63.8 (56.9, 68.8)	64.6 ± 12.2 65.0 (58.0, 72.9)	67.6 ± 11.1 69.0 (61.3, 75.2)	**<** **0.001**
Gender						**<** **0.001**
Female	794 (61.8%)	231 (54.6%)	135 (67.5%)	396 (65.9%)	32 (53.3%)	
Male	490 (38.2%)	192 (45.4%)	65 (32.5%)	205 (34.1%)	28 (46.7%)	

^a^Mean ± standard deviation.

Median (interquartile range); *n* (%).

^b^Kruskal-Wallis rank sum test; Pearson’s Chi-squared test.

**Table 2:** Baseline ocular characteristics.

Characteristic	Overall, *N* = 2002^a^	Non-Hispanic White, *N* = 653^a^	Non-Hispanic Black, *N* = 310^a^	Hispanic, *N* = 945^a^	Other, *N* = 94^a^	*p*-value^b^
Follow-up time (years)	3.2 ± 1.0 3.2 (2.4, 4.1)	3.2 ± 1.2 3.1 (2.2, 4.1)	3.3 ± 1.0 3.4 (2.5, 4.1)	3.3 ± 1.0 3.3 (2.5, 4.1)	3.1 ± 1.0 3.2 (2.4, 3.9)	0.13
Number of visits	3.8 ± 0.9 4.0 (3.0, 4.0)	3.8 ± 1.0 4.0 (3.0, 4.0)	3.7 ± 0.8 3.0 (3.0, 4.0)	3.7 ± 0.9 4.0 (3.0, 4.0)	3.8 ± 1.1 4.0 (3.0, 4.0)	0.40
Baseline HAP severity						**0.003**
Suspect	1118 (55.8%)	393 (60.2%)	152 (49.0%)	533 (56.4%)	40 (42.6%)	
Mild	440 (22.0%)	134 (20.5%)	70 (22.6%)	206 (21.8%)	30 (31.9%)	
Moderate	237 (11.8%)	58 (8.9%)	51 (16.5%)	115 (12.2%)	13 (13.8%)	
Severe	207 (10.3%)	68 (10.4%)	37 (11.9%)	91 (9.6%)	11 (11.7%)	
Baseline SAP MD (dB)	−3.01 ± 4.64 −1.76 (−3.90, −0.34)	−2.43 ± 4.13 −1.29 (−3.36, −0.03)	−3.61 ± 4.57 −2.32 (−4.45, −1.00)	−3.14 ± 4.88 −1.82 (−3.95, −0.35)	−3.86 ± 5.39 −2.37 (−4.99, −0.82)	**<** **0.001**
Baseline pRNFL thickness (µm)	82.1 ± 12.2 81.9 (73.6, 90.3)	80.4 ± 11.6 80.5 (72.1, 87.8)	83.6 ± 12.9 83.7 (74.5, 93.2)	82.9 ± 12.2 83.1 (74.7, 91.5)	80.8 ± 11.9 80.9 (70.7, 89.3)	**<** **0.001**
Baseline mGCIPL thickness (µm)	73.6 ± 9.2 74.0 (68.0, 80.0)	72.0 ± 8.9 72.0 (67.0, 78.0)	73.9 ± 10.3 74.8 (68.0, 80.9)	74.9 ± 8.8 76.0 (70.0, 81.0)	71.1 ± 9.9 72.0 (65.1, 78.0)	**<** **0.001**
CCT (µm)	546.3 ± 42.2 545.0 (519.0, 571.0)	554.2 ± 41.1 553.0 (525.0, 578.0)	530.6 ± 42.1 531.0 (506.3, 554.8)	545.4 ± 42.0 543.0 (518.0, 569.0)	552.3 ± 37.6 549.5 (531.0, 577.8)	**<** **0.001**
Mean IOP (mmHg)	15.2 ± 2.9 15.0 (13.1, 17.1)	15.3 ± 3.0 15.0 (13.3, 17.0)	15.6 ± 3.1 15.5 (13.2, 17.9)	15.2 ± 2.8 14.9 (13.0, 17.1)	14.6 ± 2.6 14.3 (12.7, 16.2)	**0.044**

^a^Mean ± standard deviation.

Median (interquartile range); *n* (%).

^b^Kruskal-Wallis rank sum test; Pearson’s Chi-squared test.

*CCT central corneal thickness, HAP Hodapp-Anderson-Parrish, IOP intraocular pressure, MD mean deviation, mGCIPL macular ganglion cell-inner plexiform layer, pRNFL peripapillary retinal nerve fibre layer, SAP standard automated perimetry.*

### Differences in pRNFL rates

Among glaucoma suspects, the mean adjusted rate of change for global pRNFL thickness was −0.44 ± 0.60 µm/year (Table 3 and Supplementary Fig. 1). A statistically significant difference among racial-ethnic groups was noted (*p* < 0.001). NHB eyes had faster rates of change in global pRNFL compared to Hispanics (−0.57 ± 0.55 µm/year vs. −0.37 ± 0.62 µm/year, respectively, *p* < 0.001), as well as in the inferior quadrant compared to NHW and Hispanics (−1.22 ± 1.03 µm/year vs. −0.79 ± 0.88 µm/year (NHW) and −0.87 ± 1.10 µm/year (Hispanic); *p* < 0.001 for both comparisons).

**Table 3:** Rates of change in peripapillary retinal nerve fibre layer thickness (µm/year) among glaucoma suspect eyes categorised by racial-ethnic group.

Characteristic	Overall, *N* = 1118^a^	Non-Hispanic White, *N* = 393^a^	Non-Hispanic Black, *N* = 152^a^	Hispanic, *N* = 533^a^	Other, *N* = 40^a^	*p*-value^b^
Number of scans	12,068	4032	1639	5982	415	
Global	−0.44 ± 0.60 −0.40 (−0.77, −0.07)	−0.49 ± 0.58 −0.44 (−0.80, −0.13)	−0.57 ± 0.55 −0.53 (−0.89, −0.25)	−0.37 ± 0.62 −0.36 (−0.72, −0.01)	−0.33 ± 0.52 −0.34 (−0.73, 0.10)	**<** **0.001**
Superior	−0.81 ± 1.18 −0.73 (−1.48, −0.09)	−0.82 ± 1.11 −0.73 (−1.48, −0.15)	−1.06 ± 1.26 −0.90 (−1.51, −0.29)	−0.75 ± 1.20 −0.65 (−1.46, −0.02)	−0.69 ± 1.05 −0.85 (−1.38, 0.18)	0.10
Inferior	−0.89 ± 1.02 −0.82 (−1.42, −0.27)	−0.79 ± 0.88 −0.76 (−1.26, −0.32)	−1.22 ± 1.03 −1.08 (−1.67, −0.56)	−0.87 ± 1.10 −0.77 (−1.47, −0.15)	−0.81 ± 0.88 −0.78 (−1.37, −0.38)	**<** **0.001**
Temporal	−0.15 ± 0.71 −0.15 (−0.46, 0.15)	−0.14 ± 0.78 −0.14 (−0.44, 0.18)	−0.21 ± 0.60 −0.20 (−0.50, 0.06)	−0.14 ± 0.69 −0.15 (−0.47, 0.15)	−0.06 ± 0.57 −0.13 (−0.43, 0.27)	0.30
Nasal	0.30 ± 0.54 0.27 (−0.03, 0.60)	0.26 ± 0.54 0.25 (−0.04, 0.56)	0.39 ± 0.55 0.36 (0.07, 0.67)	0.29 ± 0.53 0.25 (−0.02, 0.60)	0.40 ± 0.62 0.30 (0.08, 0.64)	0.058

^a^Sum; Mean ± standard deviation.

Median (interquartile range).

^b^Kruskal-Wallis rank sum test.

When evaluating eyes with mild glaucoma, the mean adjusted rate of global pRNFL was −0.52 ± 0.66 µm/year (Table 4). A significant difference in the rate of superior pRNFL quadrant change was noted (*p* = 0.022). NHB eyes demonstrated a significantly faster rate of change compared to NHW eyes in superior quadrant pRNFL (−1.20 ± 1.06 µm/year vs. −0.75 ± 1.51 µm/year, respectively; *p* = 0.043, Supplementary Fig. 1). In the inferior quadrant, NHB and Hispanic eyes tended to have faster rates compared to NHW eyes (−0.97 ± 1.32 µm/year and −1.04 ± 1.04 µm/year vs. −0.73 ± 0.97 µm/year, respectively), although this trend did not achieve statistical significance (*p* = 0.066). Global pRNFL rates tended to be faster among Hispanics (−0.59 ± 0.67 µm/year), but this also did not reach statistical significance (*p* = 0.20).

**Table 4:** Rates of change in peripapillary retinal nerve fibre layer thickness (µm/year) among mild glaucoma eyes categorised by racial-ethnic group.

Characteristic	Overall, *N* = 440^a^	Non-Hispanic White, *N* = 134^a^	Non-Hispanic Black, *N* = 70^a^	Hispanic, *N* = 206^a^	Other, *N* = 30^a^	*p*-value^b^
Number of scans	4689	1333	766	2264	326	
Global	−0.52 ± 0.66 −0.45 (−0.87, −0.12)	−0.42 ± 0.72 −0.39 (−0.80, −0.01)	−0.49 ± 0.54 −0.44 (−0.78, −0.15)	−0.59 ± 0.67 −0.54 (−0.92, −0.16)	−0.50 ± 0.48 −0.50 (−0.68, −0.13)	0.20
Superior	−0.87 ± 1.35 −0.79 (−1.66, −0.01)	−0.75 ± 1.51 −0.71 (−1.66, 0.06)	−1.20 ± 1.06 −1.08 (−1.94, −0.68)	−0.87 ± 1.34 −0.71 (−1.65, 0.00)	−0.62 ± 1.14 −0.53 (−1.17, 0.09)	**0.022**
Inferior	−0.93 ± 1.08 −0.83 (−1.48, −0.30)	−0.73 ± 0.97 −0.69 (−1.23, −0.24)	−0.97 ± 1.32 −0.78 (−1.55, −0.28)	−1.04 ± 1.04 −0.98 (−1.53, −0.38)	−0.95 ± 1.18 −0.84 (−1.82, −0.17)	0.066
Temporal	−0.21 ± 0.78 −0.22 (−0.55, 0.14)	−0.11 ± 0.80 −0.13 (−0.49, 0.18)	−0.19 ± 0.63 −0.21 (−0.46, 0.17)	−0.29 ± 0.83 −0.30 (−0.61, 0.05)	−0.10 ± 0.57 −0.09 (−0.37, 0.27)	0.062
Nasal	0.29 ± 0.49 0.27 (−0.02, 0.60)	0.33 ± 0.55 0.32 (−0.02, 0.71)	0.35 ± 0.51 0.40 (0.05, 0.65)	0.24 ± 0.44 0.22 (−0.03, 0.49)	0.28 ± 0.41 0.25 (0.03, 0.38)	0.12

^a^Sum; Mean ± standard deviation.

Median (interquartile range).

^b^Kruskal-Wallis rank sum test.

Among eyes with moderate disease, there was no significant difference in global pRNFL rate (*p* = 0.60), although NHB eyes tended to have faster rates (Table 5). There was a statistically significant difference in the rate of superior quadrant pRNFL loss (*p* = 0.006), with NHB eyes having a significantly faster rate compared to Hispanic eyes (−1.31 ± 1.49 µm/year vs. −0.52 ± 1.26 µm/year, *p* = 0.003; Table 5). No statistically significant differences were noted between racial-ethnic groups among eyes with severe glaucoma (Supplementary Table 3).

**Table 5:** Rates of change in peripapillary retinal nerve fibre layer thickness (µm/year) among moderate glaucoma eyes categorised by racial-ethnic group.

Characteristic	Overall, *N* = 237^a^	Non-Hispanic White, *N* = 58^a^	Non-Hispanic Black, *N* = 51^a^	Hispanic, *N* = 115^a^	Other, *N* = 13^a^	*p*-value^b^
Number of scans	2555	644	554	1239	118	
Global	−0.47 ± 0.68 −0.47 (−0.84, −0.03)	−0.45 ± 0.64 −0.44 (−0.95, −0.03)	−0.60 ± 0.69 −0.59 (−0.96, −0.08)	−0.42 ± 0.72 −0.40 (−0.84, −0.03)	−0.41 ± 0.47 −0.47 (−0.66, −0.02)	0.60
Superior	−0.73 ± 1.38 −0.62 (−1.50, 0.14)	−0.72 ± 1.46 −0.60 (−1.62, 0.22)	−1.31 ± 1.49 −1.28 (−2.36, −0.30)	−0.52 ± 1.26 −0.47 (−1.09, 0.20)	−0.45 ± 1.08 −0.59 (−1.13, −0.23)	**0.006**
Inferior	−0.87 ± 1.06 −0.78 (−1.45, −0.17)	−0.83 ± 1.11 −0.72 (−1.61, −0.02)	−1.07 ± 1.09 −1.04 (−1.48, −0.41)	−0.82 ± 1.05 −0.78 (−1.42, −0.14)	−0.77 ± 0.74 −0.78 (−1.15, −0.27)	0.50
Temporal	−0.22 ± 0.98 −0.18 (−0.59, 0.17)	−0.30 ± 0.94 −0.21 (−0.68, 0.12)	−0.19 ± 0.82 −0.18 (−0.66, 0.09)	−0.20 ± 1.10 −0.14 (−0.54, 0.17)	−0.25 ± 0.63 −0.39 (−0.65, 0.19)	0.90
Nasal	0.30 ± 0.56 0.27 (0.00, 0.61)	0.30 ± 0.68 0.28 (0.00, 0.50)	0.33 ± 0.57 0.32 (0.04, 0.71)	0.30 ± 0.50 0.29 (−0.03, 0.63)	0.25 ± 0.39 0.11 (0.06, 0.23)	0.70

^a^Sum; Mean ± standard deviation.

Median (interquartile range).

^b^Kruskal-Wallis rank sum test.

### Differences in mGCIPL rates

The summary of concurrent changes in mGCIPL parameters classified by racial-ethnic groups are presented in Supplementary Tables 4–7. Among glaucoma suspects, several statistically significant differences were noted between racial-ethnic groups (Supplementary Table 4). NHB eyes presented global mGCIPL rates faster than Hispanics (−0.48 ± 0.31 µm/year vs. −0.40 ± 0.29 µm/year, *p* = 0.007). In the inferotemporal sector, NHB eyes demonstrated significantly faster rates of change compared to Hispanics and NHW eyes (−0.47 ± 0.31 µm/year, vs. −0.38 ± 0.28 µm/year (Hispanic) and −0.39 ± 0.26 µm/year (NHW), *p* = 0.010 and *p* = 0.020 respectively). Statistically significant differences were also noted in the inferior and inferonasal sectors (*p* = 0.045 and *p* = 0.007 respectively), although the differences in effect sizes were small.

Among mild glaucomatous eyes, a significant difference was noted in global and inferonasal mGCIPL rates (*p* = 0.022 and *p* = 0.012 respectively). Compared to NHW eyes, Hispanic eyes had a faster global rate (−0.53 ± 0.32 µm/year vs. −0.43 ± 0.39 µm/year, *p* = 0.026; Supplementary Table 5), and inferonasal rate (−0.53 ± 0.33 µm/year vs. −0.41 ± 0.47 µm/year, *p* = 0.012; Supplementary Table 5). Among eyes with moderate disease (Supplementary Table 6), a significant difference was noted in the superotemporal sector between groups (*p* = 0.020), with NHB eyes demonstrating a faster rate of loss compared to Hispanics (−0.46 ± 0.24 µm/year vs. −0.34 ± 0.21 µm/year, *p* = 0.028). No differences were identified in rates of mGCIPL parameters among severe glaucoma eyes (*p* > 0.05 for all comparisons; Supplementary Table 7).

### Relationship between pRNFL and mGCIPL rates

We identified a strong association between global pRNFL and mGCIPL rates of change (R^2^ = 0.70). When stratified by baseline glaucoma severity (Supplementary Fig. 2), R^2^ values for relationship between global pRNFL and mGCIPL rates were consistent (glaucoma suspect: R^2^ = 0.65; mild: R^2^ = 0.72; moderate: R^2^ = 0.69; severe: R^2^ = 0.81). No differences were noted in the strength of the correlation between different racial-ethnic groups (*p* > 0.05).

## Discussion

In this study of a large treated clinical population of subjects with or suspected of glaucoma, we reported significant differences in adjusted rates of change of pRNFL and mGCIPL parameters between various racial-ethnic groups. To our knowledge, this study represents one of the largest analyses of longitudinal SD-OCT data describing both pRNFL and mGCIPL rates among a diverse population of glaucoma patients cared for in routine clinical practice. Of note, Hispanic eyes comprised almost half of the study population, a unique feature in this study. Our study identified faster rates of pRNFL and mGCIPL loss among NHB eyes across most of the spectrum of glaucomatous disease. The lack of any difference in rates among eyes with severe glaucoma may have been due to both smaller sample size and more intense treatment among these subjects. While there was a statistically significant difference in age between groups (Table 1), the estimated rates were adjusted for age as well as other key clinical parameters, such as CCT, IOP, and baseline pRNFL and mGCIPL thickness values. After accounting for these variables, NHB eyes were still noted to have significantly faster rates of pRNFL and mGCIPL loss. Hispanic eyes also tended to have faster rates among certain parameters as well, although most of these comparisons did not reach statistical significance.

Significant differences in mGCIPL rates topographically mirrored areas of significant differences in pRNFL rates in most cases. Among glaucoma suspects, we observed faster thinning in the inferior pRNFL quadrant and inferior/inferotemporal/inferonasal mGCIPL sectors in NHB eyes. Conversely, Hispanics exhibited faster rates in the inferior pRNFL quadrant and inferonasal mGCIPL sector among mild glaucoma eyes, although the former was not statistically significant. Among moderate glaucoma eyes, the superior pRNFL quadrant and supertemporal mGCIPL sector both featured faster rates among NHB eyes. These topographically correlated findings strengthen the likelihood of key differences between racial-ethnic groups.

We observed a strong association between global pRNFL and global mGCIPL rates stratified by glaucoma severity. The correlation coefficient (R^2^) between pRNFL and mGCIPL was consistent across disease stages, with slightly lower values for earlier disease (Supplementary Fig. 2). The lower correlation coefficient observed among glaucoma suspect eyes could be attributed to early changes occurring in more peripheral regions of the posterior pole, which may not have been captured by the 6x6mm GCIPL cube centred on the fovea. While counterintuitive due to proximity to the RNFL “floor”, the stronger correlation among severe eyes could be explained by our exclusion criteria; we excluded all eyes with a baseline pRNFL thickness <57 µm from the analysis, likely preventing poor correlation due to the “floor effect”. Glaucomatous loss among severe disease is likely to occur closer to the fovea, which is more likely to be captured by the Cirrus GCIPL scan.

Rates of pRNFL loss were generally greater in magnitude than those of mGCIPL, which is likely due to the differences in dynamic range of these tissues. Our finding is similar to previous studies that reported the rate of global pRNFL loss to be significantly faster than global mGCIPL loss [14, 27]. Axons from the inferotemporal, inferior, and inferonasal pGCIPL sectors all enter the optic nerve head primarily in the inferior pRNFL quadrant. Thus, pRNFL loss in this region can be thought of a composite “sum” of the respective mGCIPL losses, likely leading to a faster rate of loss when compared to just one mGCIPL sector. In addition, the Cirrus mGCIPL scan provides data only from a foveal cube, possibly missing macular damage outside of this area. However, those damaged axonal fibres would still be detected on the pRNFL scan. Prior studies have reported similar patterns of sectoral pRNFL and mGCIPL loss but in smaller cohorts of patients [13, 23, 27].

After adjusting for age, sex, CCT, IOP, and baseline thickness, we were impressed that NHB eyes still had significantly faster rates of pRNFL and mGCIPL loss. Numerous studies have demonstrated more progressive glaucomatous disease among Black patients [6, 16, 28, 29]. The Baltimore Eye Survey demonstrated that glaucoma prevalence among individuals of African descent is six times higher than that of the European American population, with a six-fold higher likelihood of glaucoma-related blindness [16]. The younger age of NHB subjects in our study (*p* < 0.001; Table 1) was also consistent with the findings of the Baltimore Eye Survey, which suggested that glaucoma tends to be diagnosed significantly earlier in the Black population. In a prospective cohort study spanning approximately 3 years and including a total of 528 healthy, glaucoma suspect, and glaucomatous eyes, Bowd et al. reported race-related differences in the rate of change of Bruch Membrane Opening-Minimum Rim Width, but only within the glaucoma suspect group [30]. No significant variation in the rate of change of pRNFL was observed. Notably, the authors did not stratify eyes with overt glaucoma according to the severity of the condition. Additionally, the study only analysed two groups (i.e., European descent and African descent), with a notable difference in the number of eyes included compared to our report. Recently, the investigators of the Primary Open-Angle African American Glaucoma Genetics (POAAGG) study described a median rate of pRNFL loss of −1.60 µm/year among Black subjects, nearly three times higher than a previously reported normal age-related rate of change of −0.52 µm/year [31]. These results are in contrast to the slower rates of pRNFL loss noted in the Duke Glaucoma Registry (−0.76 μm/year) and in the Diagnostic Innovations in Glaucoma Study (−0.98 μm/year), both which were predominantly comprised of White subjects (67.8% and 67%, respectively) [8, 14]. It is important to note that the POAAGG study had a non-comparative design, focusing solely on African American, African descent, or African Caribbean patients. Direct comparisons with other racial and ethnic groups were not made in the study, and the authors did not analyse mGCIPL data, sectoral pRNFL data, or stratify rates based on glaucomatous severity [31]. With respect to rates of OCT parameters among Hispanic patients, we believe that our study represents the first report documenting pRNFL and mGCIPL rates in a large clinical cohort.

Aetiologies for the faster rates of loss among NHB subjects could be related to different factors. A major strength of studying a clinically treated population is analysing the potential impact of non-clinical parameters on testing results, such as poor medication adherence, which are often non-issues in prospective studies due to exclusion criteria. Socioeconomic disparities and differences in access to healthcare have been described to contribute to differences in glaucoma outcomes among racial and ethnic groups [26, 32]. Black patients are more likely to face barriers to care such as lack of insurance, transportation difficulties, limited access to medications, as well as lower adherence to follow-up and treatment regimens, which can negatively impact health outcomes [26, 32, 33]. These disparities, coupled with worse baseline disease at diagnosis and at a younger age, could contribute to faster rates of pRNFL and mGCIPL loss, especially in earlier disease as observed in this study [26, 32]. In contrast, treatment adherence or surgical intervention might be greater in severe glaucoma given the perceptible impact on vision, leading to a non-significant difference in rates (Supplementary Table 3). Addressing these socioeconomic challenges may potentially ameliorate the disparities observed in this study, although further analyses are required as we were unable to distinguish between potential social influences. In addition, genetic differences could predispose NHB eyes to rapid disease progression; for example, the POAAGG study identified potential genetic factors that could contribute to worse disease among Black patients, specifically the TC genotype in a single nuclear polymorphism near the TRIM66 gene [31, 34]. In our study, CCT was significantly lower among NHB eyes (Table 2), potentially suggesting a difference in sclera and lamina cribrosa biomechanics, which may dictate how elevated IOP or IOP fluctuations impact pRNFL and mGCIPL loss. These findings highlight the complex interplay of genetic, ocular, and socioenvironmental factors that may influence the progression of glaucoma and contribute to the observed disparities between racial-ethnic groups [35].

Strengths of this analysis include the study cohort, which consists of a large, clinically treated population with considerable follow-up. Of note, our study population was unique given the substantial representation of Hispanic subjects, accounting for nearly 50%. In addition, the statistical modelling approach of longitudinal joint mixed modelling in this study minimises the degree of measurement error by allowing two associated outcomes to be modelled together. This study was also unique in its modelling of topographically related sections of pRNFL quadrants and mGCIPL sectors. Furthermore, excluding tests conducted after any surgical procedure carried out during the follow-up period enabled a more conservative analysis of the rate of pRNFL or mGCIPL loss, reducing the influence of iatrogenic factors.

Use of EHR data has its inherent limitations, including incorrect ICD or CPT coding. The diagnosis of glaucoma or glaucoma suspect was made based on the clinician’s judgement. In addition, any glaucoma surgeries that occurred outside of BPEI would not have been accounted for when excluding post-intervention tests. Furthermore, certain trends were observed in the data as previously noted, but these differences did not reach statistical significance in our current sample. These trends may represent true differences that would reach statistical significance with a larger sample size. Finally, our clinical population did not have sufficiently large cohorts of other racial-ethnic groups (e.g., Asian or Native American), limiting our conclusions regarding these individuals.

In summary, our analysis of a treated clinical glaucomatous population highlights significant differences in pRNFL and mGCIPL rates across different racial-ethnic groups after stratifying by disease severity. Rates of mGCIPL loss mirrored those of pRNFL, with non-Hispanic Black eyes having faster rates of pRNFL and mGCIPL loss among glaucoma suspect, mild glaucoma, and moderate glaucoma eyes. These findings reflect the importance of considering various aetiologies, such as socioeconomic disparities, that may contribute to these differences in rates of OCT loss among glaucoma patients.

Supplemental material is available at Eye’s website.

## Summary

### What was known before

- Differences in rates of change in OCT parameters between diverse racial-ethnic groups have not been investigated using a large population receiving routine care.

### What this study adds

- In this retrospective cohort study including 2002 eyes, non-Hispanic Black eyes featured the highest rates of peripapillary Retinal Nerve Fibre Layer (pRNFL) loss among glaucoma suspect, mild, and moderate glaucoma eyes in a treated clinical population. Global pRNFL and macular Ganglion Cell Inner Plexiform Layer rates were strongly correlated. Racial-ethnic differences must be considered in the management of glaucoma patients, given the potential for a more rapid rate of disease progression within certain groups.

## Supplementary information

Supplementary Figure 1

Supplementary Figure 2

Supplemental Tables

Supplementary Figure Legends

## Data Availability

The data that support the findings of this study are available from the authors upon reasonable request.
