# Supplementary figures and images for: Racial-ethnic disparities in concurrent rates of peripapillary & macular OCT parameters among a large glaucomatous clinical population
Source: Eye (Lond). 2024 May 4;38(14):2711–7. doi: 10.1038/s41433-024-03103-3 (PMC11427570; doi:10.1038/s41433-024-03103-3)

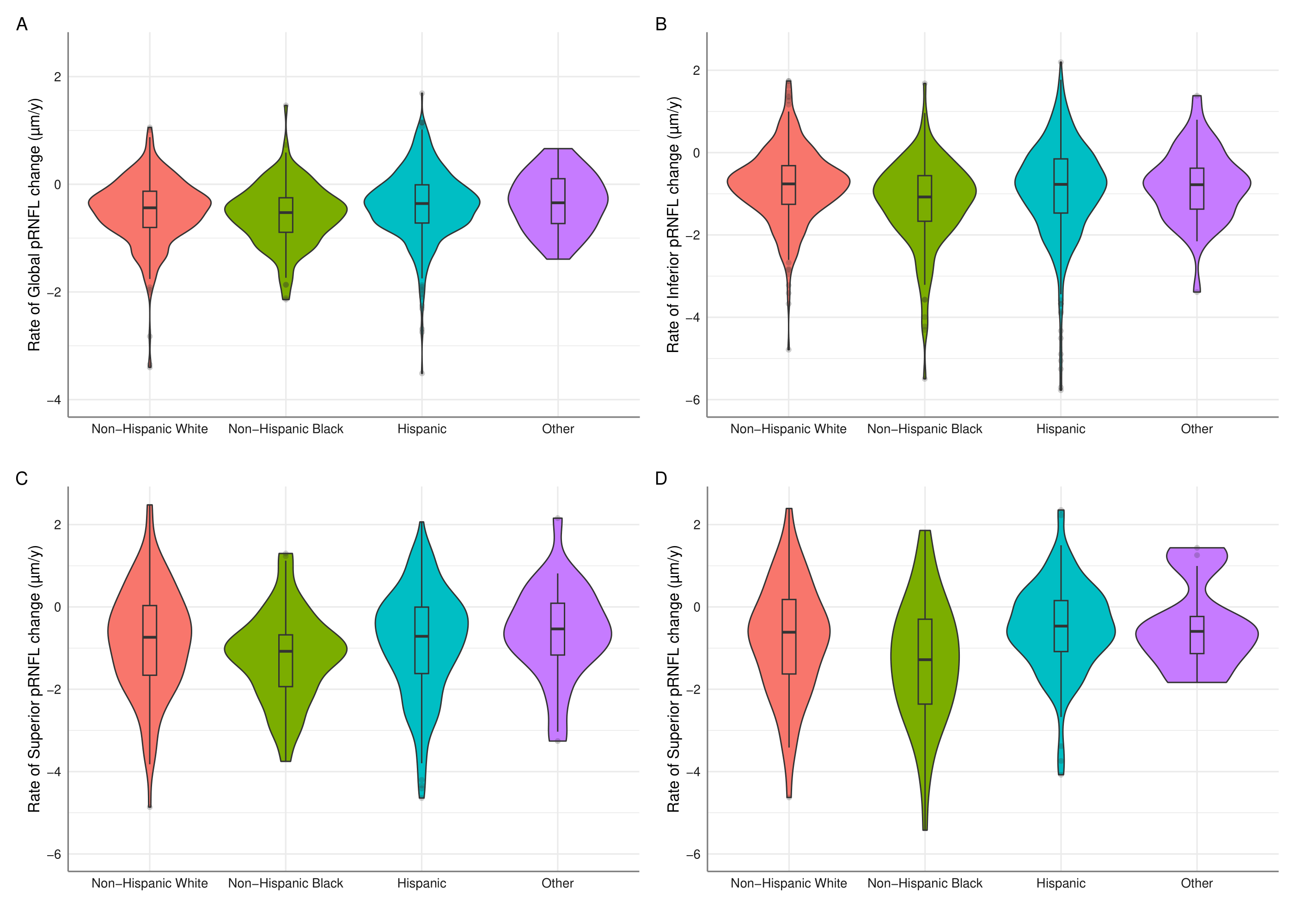

Supplement: Supplementary file 1 — Supplementary Figure 1 [file 41433_2024_3103_MOESM1_ESM.tif]

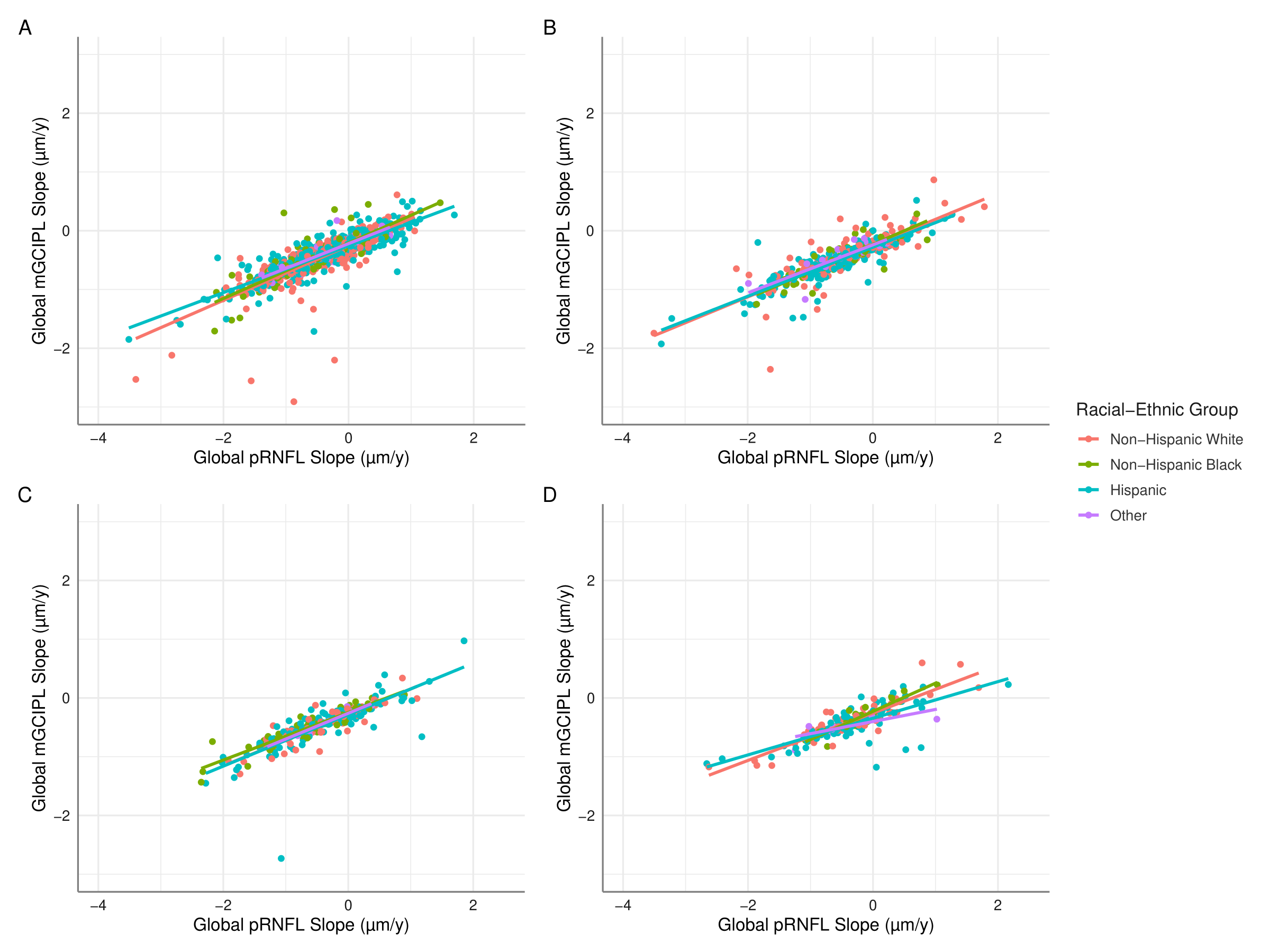

Supplement: Supplementary file 2 — Supplementary Figure 2 [file 41433_2024_3103_MOESM2_ESM.tif]
