# Supplementary material for: Racial-ethnic disparities in concurrent rates of peripapillary & macular OCT parameters among a large glaucomatous clinical population
Source: Eye (Lond). 2024 May 4;38(14):2711–7. doi: 10.1038/s41433-024-03103-3 (PMC11427570; doi:10.1038/s41433-024-03103-3)
Supplement: Supplementary file 3 — Supplemental Tables [file 41433_2024_3103_MOESM3_ESM.docx]

Supplementary Table 1. List of International Classification of Diseases codes used to identify patients with or suspected of glaucoma.

| **Inclusion ICD Codes** | | |
| --- | --- | --- |
|  | **ICD-9** | **ICD-10** |
| **Glaucoma suspect** | 365.01, 365.02, 365.03, 365.04, 365.05, 365.06  377.14^a^  V19.11^b^ | H40.00X, H40.01X, H40.02X, H40.03X, H40.04X, H40.05X, H40.06X  H47.23X^a^  Z83.511^b^ |
| **Open angle glaucoma** | 365.10  365.11  365.12  365.13  365.14  365.15  365.52  365.7X | H40.1X  Q15.0 |
| **Chronic angle closure glaucoma (excluding acute angle closure)** | 365.20, 365.21, 365.23, 365.24 | H40.22X, H40.23X, H40.24X |
| **Traumatic glaucoma** | 365.65 | H40.3X |
| **Uveitic glaucoma** | 365.62 | H40.4X |
| **Glaucoma due to other disorders** | 365.63 | H40.5X |
| **Drug-induced glaucoma** | 365.31, 365.32 | H40.6X |
| **Episcleral venous pressure-induced glaucoma, hypersecretion glaucoma, malignant glaucoma** | 365.81, 365.82, 365.83 | H40.8X |

*ICD: International Classification of Diseases*

*X replaces any combination of numbers*

^a^ Glaucomatous optic disc atrophy; in the absence of other ICD codes for glaucoma.

^b^ Family history of glaucoma; in the absence of other ICD codes for glaucoma.

Supplementary Table 2. List of exclusionary International Classification of Diseases codes.

| **Exclusion ICD Codes** | | |
| --- | --- | --- |
|  | **ICD-9** | **ICD-10** |
| **Age-related macular degeneration** | 362.5X | H35.31X^a^  H35.32X |
| **Amblyopia** | 368.0X | H53.0X |
| **Intraocular tumors** | 190.5X; 190.6X | C69.2X; C69.3X |
| **Optical neuritis and optic nerve and visual pathways diseases** | 377.X, except 377.14^b^ | H46.X  H47.X, except H47.23X^b^ |
| **Retinal detachment** | 361.0X; 361.2X; 361.8X; 361.9X | H33.0X; H33.2X; H33.4X |
| **Retinal vascular occlusions and ischemia** | 362.3X  362.84 | H34.X  H35.82 |
| **Uveitis and endophthalmitis** | 360.0X; 360.1X  361.X  363.X  364.0X; 364.1X; 364.2X; 364.3X | H20.02X  H30.X  H44.0X; H44.1X |
| **Proliferative diabetic retinopathy** | 362.02 | E10.35X  E11.35X |
| **Other retinopathies** | 362.1X, 362.2X | H35.0X, H35.7X, H35.8X |
| **Retinal dystrophies** | 362.7X | H35.1X, H35.5X |

*ICD: International Classification of Diseases*

*X replaces any combination of numbers*

^a^ Advanced and atrophic dry stages only.

^b^ Glaucomatous optic disc atrophy.

Supplementary Table 3. Rates of change in peripapillary retinal nerve fiber layer thickness (µm/year) among severe glaucoma eyes categorized by racial-ethnic group.

| **Characteristic** | **Overall**, N = 207^1^ | **Non-Hispanic White**, N = 68^1^ | **Non-Hispanic Black**, N = 37^1^ | **Hispanic**, N = 91^1^ | **Other**, N = 11^1^ | **p-value**^2^ |
| --- | --- | --- | --- | --- | --- | --- |
| Number of scans | 2,160 | 719 | 378 | 937 | 126 |  |
| Global | -0.45 ± 0.89  -0.42 (-0.75, -0.06) | -0.50 ± 0.83  -0.52 (-0.83, -0.13) | -0.30 ± 0.45  -0.35 (-0.53, -0.08) | -0.46 ± 1.08  -0.40 (-0.72, 0.01) | -0.47 ± 0.65  -0.65 (-0.91, -0.04) | 0.40 |
| Superior | -0.70 ± 1.30  -0.69 (-1.27, -0.03) | -0.83 ± 1.30  -0.76 (-1.31, -0.09) | -0.43 ± 1.08  -0.57 (-1.01, 0.03) | -0.73 ± 1.36  -0.50 (-1.29, -0.03) | -0.45 ± 1.44  -0.94 (-1.35, 0.31) | 0.70 |
| Inferior | -0.91 ± 1.65  -0.73 (-1.21, -0.27) | -0.96 ± 2.19  -0.74 (-1.11, -0.32) | -0.76 ± 0.75  -0.81 (-1.11, -0.25) | -0.93 ± 1.54  -0.64 (-1.34, -0.27) | -1.04 ± 0.92  -1.18 (-1.76, -0.25) | 0.80 |
| Temporal | -0.19 ± 1.12  -0.24 (-0.60, 0.18) | -0.09 ± 1.28  -0.30 (-0.57, 0.10) | -0.22 ± 0.80  -0.21 (-0.62, 0.41) | -0.24 ± 1.14  -0.25 (-0.61, 0.11) | -0.33 ± 1.03  -0.19 (-0.87, 0.28) | >0.9 |
| Nasal | 0.24 ± 0.53  0.23 (-0.04, 0.52) | 0.34 ± 0.52  0.32 (0.09, 0.62) | 0.21 ± 0.54  0.16 (-0.12, 0.37) | 0.20 ± 0.53  0.23 (-0.05, 0.45) | 0.10 ± 0.51  0.22 (-0.25, 0.43) | 0.11 |
| ^1^Sum; Mean ± SD  Median (IQR) | | | | | | |
| ^2^Kruskal-Wallis rank sum test | | | | | | |

Supplementary Table 4. Rates of change in macular ganglion cell-inner plexiform layer thickness (µm/year) among glaucoma suspects categorized by racial-ethnic group.

| **Characteristic** | **Overall**, N = 1,118^1^ | **Non-Hispanic White**, N = 393^1^ | **Non-Hispanic Black**, N = 152^1^ | **Hispanic**, N = 533^1^ | **Other**, N = 40^1^ | **p-value**^2^ |
| --- | --- | --- | --- | --- | --- | --- |
| Number of scans | 8,090 | 2,830 | 1,103 | 3,859 | 298 |  |
| Global | -0.44 ± 0.33  -0.42 (-0.58, -0.27) | -0.49 ± 0.40  -0.44 (-0.60, -0.30) | -0.48 ± 0.31  -0.47 (-0.63, -0.31) | -0.40 ± 0.29  -0.40 (-0.54, -0.24) | -0.36 ± 0.25  -0.35 (-0.56, -0.18) | **<0.001** |
| Minimum | -0.65 ± 0.96  -0.56 (-0.80, -0.32) | -0.72 ± 1.27  -0.54 (-0.79, -0.31) | -0.67 ± 0.63  -0.61 (-0.86, -0.39) | -0.62 ± 0.76  -0.56 (-0.81, -0.34) | -0.30 ± 0.80  -0.48 (-0.64, -0.31) | 0.078 |
| Superotemporal | -0.36 ± 0.18  -0.34 (-0.45, -0.26) | -0.37 ± 0.17  -0.36 (-0.46, -0.27) | -0.36 ± 0.19  -0.34 (-0.44, -0.25) | -0.35 ± 0.18  -0.33 (-0.44, -0.24) | -0.34 ± 0.15  -0.34 (-0.41, -0.28) | 0.20 |
| Inferotemporal | -0.39 ± 0.28  -0.37 (-0.53, -0.23) | -0.39 ± 0.26  -0.35 (-0.51, -0.24) | -0.47 ± 0.31  -0.41 (-0.60, -0.28) | -0.38 ± 0.28  -0.37 (-0.53, -0.20) | -0.37 ± 0.24  -0.38 (-0.51, -0.22) | **0.015** |
| Superior | -0.45 ± 0.33  -0.41 (-0.59, -0.27) | -0.48 ± 0.36  -0.45 (-0.60, -0.29) | -0.41 ± 0.38  -0.38 (-0.55, -0.23) | -0.44 ± 0.29  -0.40 (-0.58, -0.27) | -0.42 ± 0.32  -0.44 (-0.58, -0.22) | 0.15 |
| Inferior | -0.43 ± 0.41  -0.38 (-0.57, -0.24) | -0.47 ± 0.47  -0.39 (-0.58, -0.26) | -0.48 ± 0.39  -0.44 (-0.64, -0.26) | -0.40 ± 0.34  -0.37 (-0.55, -0.22) | -0.29 ± 0.59  -0.34 (-0.63, -0.14) | **0.045** |
| Superonasal | -0.44 ± 0.29  -0.44 (-0.58, -0.30) | -0.44 ± 0.32  -0.44 (-0.56, -0.32) | -0.48 ± 0.27  -0.48 (-0.61, -0.31) | -0.44 ± 0.28  -0.44 (-0.59, -0.30) | -0.40 ± 0.31  -0.44 (-0.50, -0.22) | 0.30 |
| Inferonasal | -0.51 ± 0.37  -0.51 (-0.66, -0.34) | -0.52 ± 0.41  -0.49 (-0.64, -0.32) | -0.55 ± 0.38  -0.57 (-0.71, -0.40) | -0.50 ± 0.35  -0.52 (-0.66, -0.35) | -0.43 ± 0.26  -0.39 (-0.60, -0.24) | **0.007** |
| ^1^Sum; Mean ± SD  Median (IQR) | | | | | | |
| ^2^Kruskal-Wallis rank sum test | | | | | | |

Supplementary Table 5. Rates of change in macular ganglion cell-inner plexiform layer thickness (µm/year) among mild glaucoma eyes categorized by racial-ethnic group.

| **Characteristic** | **Overall**, N = 440^1^ | **Non-Hispanic White**, N = 134^1^ | **Non-Hispanic Black**, N = 70^1^ | **Hispanic**, N = 206^1^ | **Other**, N = 30^1^ | **p-value**^2^ |
| --- | --- | --- | --- | --- | --- | --- |
| Number of scans | 3,182 | 953 | 487 | 1,517 | 225 |  |
| Global | -0.48 ± 0.33  -0.46 (-0.63, -0.30) | -0.43 ± 0.39  -0.42 (-0.61, -0.25) | -0.46 ± 0.29  -0.44 (-0.61, -0.29) | -0.53 ± 0.32  -0.50 (-0.66, -0.33) | -0.44 ± 0.23  -0.43 (-0.55, -0.28) | **0.022** |
| Minimum | -0.71 ± 1.13  -0.59 (-0.91, -0.32) | -0.62 ± 1.41  -0.52 (-0.85, -0.24) | -0.83 ± 1.47  -0.64 (-0.86, -0.45) | -0.74 ± 0.80  -0.62 (-0.93, -0.34) | -0.68 ± 0.70  -0.54 (-0.86, -0.32) | 0.20 |
| Superotemporal | -0.39 ± 0.22  -0.39 (-0.50, -0.24) | -0.38 ± 0.25  -0.38 (-0.49, -0.24) | -0.42 ± 0.17  -0.40 (-0.53, -0.31) | -0.40 ± 0.21  -0.38 (-0.51, -0.25) | -0.31 ± 0.18  -0.30 (-0.41, -0.18) | 0.057 |
| Inferotemporal | -0.47 ± 0.31  -0.44 (-0.64, -0.28) | -0.45 ± 0.31  -0.43 (-0.62, -0.26) | -0.42 ± 0.32  -0.41 (-0.55, -0.23) | -0.50 ± 0.30  -0.47 (-0.69, -0.30) | -0.43 ± 0.35  -0.40 (-0.64, -0.27) | 0.12 |
| Superior | -0.47 ± 0.33  -0.45 (-0.67, -0.28) | -0.50 ± 0.36  -0.44 (-0.69, -0.29) | -0.41 ± 0.29  -0.37 (-0.58, -0.22) | -0.48 ± 0.33  -0.47 (-0.67, -0.29) | -0.38 ± 0.28  -0.39 (-0.54, -0.14) | 0.14 |
| Inferior | -0.51 ± 0.45  -0.46 (-0.72, -0.23) | -0.51 ± 0.48  -0.47 (-0.70, -0.23) | -0.45 ± 0.49  -0.40 (-0.57, -0.20) | -0.54 ± 0.42  -0.46 (-0.74, -0.24) | -0.49 ± 0.46  -0.46 (-0.71, -0.23) | 0.30 |
| Superonasal | -0.41 ± 0.32  -0.42 (-0.58, -0.26) | -0.35 ± 0.38  -0.40 (-0.56, -0.22) | -0.45 ± 0.23  -0.45 (-0.57, -0.32) | -0.43 ± 0.30  -0.43 (-0.59, -0.28) | -0.40 ± 0.34  -0.39 (-0.59, -0.30) | 0.40 |
| Inferonasal | -0.49 ± 0.37  -0.51 (-0.67, -0.33) | -0.41 ± 0.47  -0.42 (-0.64, -0.23) | -0.54 ± 0.22  -0.56 (-0.66, -0.39) | -0.53 ± 0.33  -0.55 (-0.69, -0.37) | -0.53 ± 0.33  -0.44 (-0.68, -0.34) | **0.012** |
| ^1^Sum; Mean ± SD  Median (IQR) | | | | | | |
| ^2^Kruskal-Wallis rank sum test | | | | | | |

Supplementary Table 6. Rates of change in macular ganglion cell-inner plexiform layer thickness (µm/year) among moderate glaucoma eyes categorized by racial-ethnic group.

| **Characteristic** | **Overall**, N = 237^1^ | **Non-Hispanic White**, N = 58^1^ | **Non-Hispanic Black**, N = 51^1^ | **Hispanic**, N = 115^1^ | **Other**, N = 13^1^ | **p-value**^2^ |
| --- | --- | --- | --- | --- | --- | --- |
| Number of scans | 1,735 | 453 | 365 | 825 | 92 |  |
| Global | -0.47 ± 0.35  -0.44 (-0.64, -0.29) | -0.48 ± 0.31  -0.45 (-0.67, -0.25) | -0.49 ± 0.30  -0.47 (-0.65, -0.31) | -0.47 ± 0.40  -0.43 (-0.60, -0.30) | -0.44 ± 0.21  -0.49 (-0.60, -0.22) | >0.9 |
| Minimum | -0.69 ± 1.01  -0.61 (-0.90, -0.31) | -0.72 ± 1.03  -0.57 (-0.84, -0.30) | -0.77 ± 0.57  -0.65 (-1.03, -0.44) | -0.65 ± 1.19  -0.61 (-0.90, -0.28) | -0.53 ± 0.43  -0.57 (-0.80, -0.17) | 0.40 |
| Superotemporal | -0.37 ± 0.23  -0.35 (-0.51, -0.23) | -0.36 ± 0.24  -0.36 (-0.52, -0.17) | -0.46 ± 0.24  -0.44 (-0.63, -0.30) | -0.34 ± 0.21  -0.34 (-0.46, -0.23) | -0.29 ± 0.22  -0.31 (-0.40, -0.16) | **0.020** |
| Inferotemporal | -0.52 ± 0.36  -0.49 (-0.72, -0.29) | -0.60 ± 0.40  -0.55 (-0.85, -0.32) | -0.54 ± 0.38  -0.50 (-0.70, -0.30) | -0.47 ± 0.33  -0.46 (-0.66, -0.27) | -0.48 ± 0.33  -0.37 (-0.83, -0.30) | 0.30 |
| Superior | -0.44 ± 0.41  -0.43 (-0.67, -0.24) | -0.42 ± 0.42  -0.43 (-0.61, -0.19) | -0.49 ± 0.40  -0.43 (-0.76, -0.25) | -0.43 ± 0.41  -0.44 (-0.65, -0.26) | -0.35 ± 0.39  -0.39 (-0.58, -0.03) | 0.70 |
| Inferior | -0.57 ± 0.54  -0.52 (-0.81, -0.28) | -0.61 ± 0.60  -0.50 (-0.84, -0.24) | -0.45 ± 0.51  -0.50 (-0.69, -0.29) | -0.60 ± 0.54  -0.53 (-0.82, -0.34) | -0.51 ± 0.37  -0.52 (-0.82, -0.26) | 0.70 |
| Superonasal | -0.36 ± 0.29  -0.39 (-0.53, -0.22) | -0.29 ± 0.30  -0.33 (-0.46, -0.16) | -0.41 ± 0.23  -0.43 (-0.55, -0.27) | -0.37 ± 0.31  -0.40 (-0.53, -0.23) | -0.42 ± 0.26  -0.36 (-0.59, -0.25) | 0.10 |
| Inferonasal | -0.48 ± 0.38  -0.48 (-0.65, -0.28) | -0.46 ± 0.34  -0.44 (-0.63, -0.28) | -0.44 ± 0.31  -0.50 (-0.64, -0.21) | -0.50 ± 0.45  -0.49 (-0.67, -0.32) | -0.58 ± 0.22  -0.60 (-0.68, -0.48) | 0.40 |
| ^1^Sum; Mean ± SD  Median (IQR) | | | | | | |
| ^2^Kruskal-Wallis rank sum test | | | | | | |

Supplementary Table 7. Rates of change in macular ganglion cell-inner plexiform layer thickness (µm/year) among severe glaucoma eyes categorized by racial-ethnic group.

| **Characteristic** | **Overall**, N = 207^1^ | **Non-Hispanic White**, N = 68^1^ | **Non-Hispanic Black**, N = 37^1^ | **Hispanic**, N = 91^1^ | **Other**, N = 11^1^ | **p-value**^2^ |
| --- | --- | --- | --- | --- | --- | --- |
| Number of scans | 1,424 | 501 | 252 | 596 | 75 |  |
| Global | -0.47 ± 0.46  -0.47 (-0.58, -0.29) | -0.46 ± 0.35  -0.48 (-0.57, -0.30) | -0.37 ± 0.23  -0.40 (-0.52, -0.26) | -0.52 ± 0.60  -0.47 (-0.61, -0.32) | -0.50 ± 0.18  -0.50 (-0.57, -0.35) | 0.30 |
| Minimum | -0.62 ± 0.80  -0.61 (-0.80, -0.43) | -0.59 ± 0.58  -0.56 (-0.79, -0.41) | -0.51 ± 0.38  -0.61 (-0.73, -0.47) | -0.69 ± 1.07  -0.64 (-0.83, -0.43) | -0.52 ± 0.36  -0.57 (-0.74, -0.35) | 0.60 |
| Superotemporal | -0.37 ± 0.23  -0.38 (-0.48, -0.25) | -0.40 ± 0.22  -0.40 (-0.49, -0.29) | -0.30 ± 0.21  -0.35 (-0.44, -0.19) | -0.38 ± 0.25  -0.36 (-0.48, -0.24) | -0.33 ± 0.24  -0.38 (-0.46, -0.26) | 0.40 |
| Inferotemporal | -0.59 ± 0.40  -0.54 (-0.83, -0.33) | -0.61 ± 0.40  -0.55 (-0.86, -0.34) | -0.58 ± 0.31  -0.50 (-0.81, -0.31) | -0.58 ± 0.45  -0.52 (-0.78, -0.32) | -0.64 ± 0.35  -0.56 (-0.94, -0.37) | 0.80 |
| Superior | -0.43 ± 0.39  -0.45 (-0.63, -0.25) | -0.43 ± 0.34  -0.46 (-0.63, -0.31) | -0.38 ± 0.36  -0.41 (-0.56, -0.19) | -0.45 ± 0.44  -0.43 (-0.63, -0.23) | -0.37 ± 0.45  -0.33 (-0.52, -0.12) | 0.70 |
| Inferior | -0.64 ± 0.64  -0.49 (-0.90, -0.27) | -0.67 ± 0.66  -0.51 (-1.02, -0.33) | -0.58 ± 0.54  -0.47 (-0.95, -0.20) | -0.64 ± 0.70  -0.49 (-0.85, -0.27) | -0.56 ± 0.44  -0.37 (-0.81, -0.31) | >0.9 |
| Superonasal | -0.29 ± 0.35  -0.33 (-0.48, -0.12) | -0.27 ± 0.34  -0.33 (-0.47, -0.10) | -0.29 ± 0.27  -0.29 (-0.48, -0.05) | -0.31 ± 0.39  -0.34 (-0.49, -0.14) | -0.24 ± 0.30  -0.33 (-0.41, -0.11) | >0.9 |
| Inferonasal | -0.46 ± 0.41  -0.48 (-0.61, -0.32) | -0.42 ± 0.42  -0.48 (-0.61, -0.27) | -0.44 ± 0.34  -0.50 (-0.66, -0.30) | -0.50 ± 0.44  -0.48 (-0.65, -0.32) | -0.40 ± 0.29  -0.43 (-0.58, -0.38) | >0.9 |
| ^1^Sum; Mean ± SD  Median (IQR) | | | | | | |
| ^2^Kruskal-Wallis rank sum test | | | | | | |
