# Supplementary material for: Racial-ethnic disparities in concurrent rates of peripapillary & macular OCT parameters among a large glaucomatous clinical population
Source: Eye (Lond). 2024 May 4;38(14):2711–7. doi: 10.1038/s41433-024-03103-3 (PMC11427570; doi:10.1038/s41433-024-03103-3)
Supplement: Supplementary file 4 — Supplementary Figure Legends [file 41433_2024_3103_MOESM4_ESM.docx]

**Supplementary Figure Legends**

**Supplementary Figure 1**. Violin plots comparing the rates of peripapillary retinal nerve fibre layer (pRNFL) loss among different racial-ethnic backgrounds: rate of (A) global pRNFL among glaucoma suspect eyes, (B) inferior pRNFL quadrant among glaucoma suspect eyes, (C) superior pRNFL quadrant among mild glaucoma eyes, and (D) superior pRNFL quadrant among moderate glaucoma eyes.

**Supplementary Figure 2**. Comparison of global peripapillary retinal nerve fibre layer (pRNFL) and global macular ganglion cell-inner plexiform layer (mGCIPL) rates of change classified by racial-ethnic group among (A) glaucoma suspect, (B) mild, (C) moderate, and (D) severe glaucoma.
